# Alternative endoscopic ultrasound-guided choledochogastrostomy for anatomical inaccessibility by usual biliary drainage methods

**DOI:** 10.1055/a-2344-8116

**Published:** 2024-07-15

**Authors:** Hiroaki Tsuji, Ryota Sagami, Takao Sato, Hidefumi Nishikiori, Yasuhisa Hiroshima, Kazuhiro Mizukami, Kazunari Murakami

**Affiliations:** 1 157533 Department of Gastroenterology, Oita San-ai Medical Center, Oita, Japan; 2 Department of Gastroenterology, Oita University, Faculty of Medicine, Yufu, Japan

A 92-year-old man was admitted with jaundice and cholangitis. Computed tomography (CT) and magnetic resonance cholangiopancreatography (MRCP) revealed intrahepatic/middle bile duct dilatation, with a pancreatic head lesion suspicious for carcinoma ( Fig. 1 **a, c** ); the intraperitoneal organs were drawn into the right thoracic cavity due to Chilaiditi syndrome ( Fig. 1 **b** ). Despite wire-guided insertion, endoscopic retrograde cholangiography (ERC) could not reach the papilla of Vater owing to anatomical abnormalities ( Fig. 2 ). Endoscopic ultrasound-guided hepaticogastrostomy failed because of invisibility of the left intrahepatic duct. The common bile duct (CBD), which in normal anatomy is not clearly visualized from the gastric tract, could be visualized from the antrum. Endoscopic ultrasound-guided choledochogastrostomy (EUS-CGS) was ultimately performed ( Video 1 ).

**Fig. 1:**
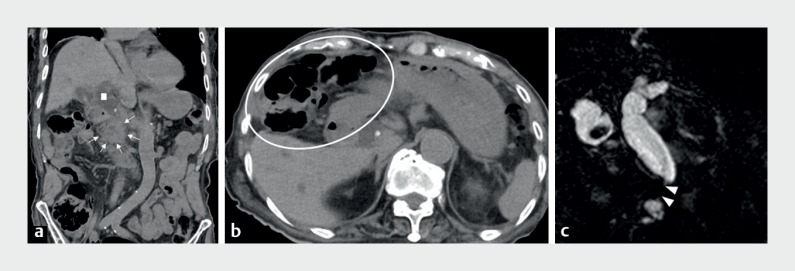
Imaging of a pancreatic head carcinoma in a patient with Chilaiditi syndrome showing on: **a, b** on computed tomography images, dilatation of the extrahepatic bile duct (white square) and a pancreatic head carcinoma (white arrows), with the intraperitoneal organs drawn into the right thoracic cavity and the jejunum lying in front of the liver (white circle) owing to Chilaiditi syndrome **c** on magnetic resonance cholangiopancreatography, severe lower bile duct stenosis (arrow heads).

**Fig. 2:**
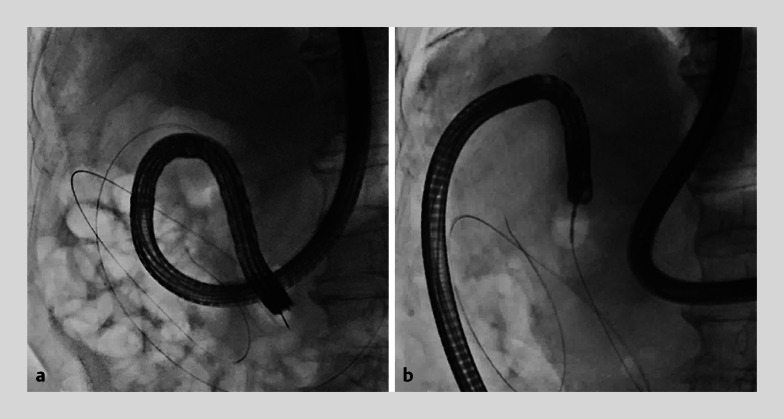
Endoscopic imaging showing that the upper gastrointestinal scope and duodenoscope could not be passed into the descending duodenum, despite wire-guided insertion, because of the anatomical abnormalities.

Endoscopic ultrasound-guided choledochogastrostomy was performed in a patient with pancreatic head carcinoma and anatomical inaccessibility to the papilla of Vater due to Chilaiditi syndrome.Video 1

Using an echoendoscope (UCT260; Olympus Medical Systems, Tokyo, Japan) connected to ultrasound (Aloka Alietta 850; Hitachi Medical System, Tokyo, Japan), we punctured the CBD via the antrum using a 19-gauge needle and Doppler echo mode to avoid blood vessels ( Fig. 3 **a** ). A 0.025-inch guidewire was introduced upside down ( Fig. 3 **b** ). An 8-mm × 8-cm fully-covered self-expandable metal stent (Hanaro Benefit; Boston Scientific, Tokyo, Japan) was deployed from the antrum to the CBD, without mechanical dilation ( Fig. 3 **c** ). The patient’s cholangitis and jaundice rapidly improved and he was discharged from the hospital 7 days postoperatively. After 2 months, the stent remained in place, despite cancerous ascites having become apparent on CT. The patient died of his primary disease 143 days postoperatively, without having experienced recurrent jaundice.

**Fig. 3:**
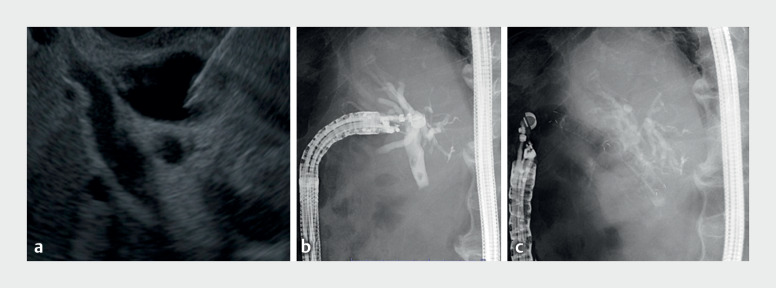
Images during endoscopic ultrasound-guided choledochogastrostomy showing: **a** the common bile duct being punctured from the antrum using a 19-gauge needle; **b** contrast medium being injected into the common bile duct, with a 0.025-inch guidewire introduced upside down in comparison to a normal choledochoduodenostomy; **c** an 8-mm × 8-cm fully-covered self-expandable metal stent in position between the antrum and the common bile duct.

EUS-guided biliary drainage is an alternative for failed ERC, with similar safety and efficacy 1 2 3 ; however, endoscopic procedures require careful attention in patients with Chilaiditi syndrome, which has a prevalence of 0.025%–0.28% of the general population 4 . This is the first report of EUS-CGS being performed in a patient with Chilaiditi syndrome. Acrobatic EUS-CGS should be considered for patients with anatomical abnormalities, for whom standard drainage is not possible.

Endoscopy_UCTN_Code_TTT_1AS_2AH

## Notes

Endoscopy E-Videos https://eref.thieme.de/e-videos
*E-Videos* is an open access online section of the journal *Endoscopy* , reporting on interesting cases and new techniques in gastroenterological endoscopy. All papers include a high-quality video and are published with a Creative Commons CC-BY license. Endoscopy E-Videos qualify for HINARI discounts and waivers and eligibility is automatically checked during the submission process. We grant 100% waivers to articles whose corresponding authors are based in Group A countries and 50% waivers to those who are based in Group B countries as classified by Research4Life (see: https://www.research4life.org/access/eligibility/ ). This section has its own submission website at https://mc.manuscriptcentral.com/e-videos .
